# Leaf *versus* flower: green-synthesized silver nanoparticles from *Xanthoceras sorbifolia* leaf extract reveal superior antimicrobial and cytotoxic efficacy

**DOI:** 10.1039/d5ra02434j

**Published:** 2025-07-08

**Authors:** Yongqiang Han, Renchi Fu, Yanyang Dai, Chao Tan, Wenjie Wang, Dongdong Guo, Zhongling Ma, Xiaojun Zhang

**Affiliations:** a Northwest University Chang An Hospital, Northwest University Xi'an Shaanxi 710069 China; b School of Medicine, Northwest University Xi'an Shaanxi 710069 China zhangxj@nwu.edu.cn; c Key Laboratory of Resource Biology and Biotechnology Western China, Ministry of Education, Provincial Key Laboratory of Biotechnology, College of Life Sciences, Northwest University Xi'an Shaanxi 710069 China; d Northwest University Chang An Hospital, Faculty of Life Sciences and Medicine, Northwest University Xi'an Shaanxi 710069 China mazhongling0524@163.com; e Department of Oncology, Chang An District Hospital Xi'an Shaanxi 710118 China

## Abstract

Green-synthesized silver nanoparticles (AgNPs) have emerged as promising antimicrobial agents, yet optimizing their synthesis and understanding their biological mechanisms remain crucial challenges. This study reports the synthesis of AgNPs using *Xanthoceras sorbifolia* leaf and flower extracts, leveraging their phytochemical composition for green synthesis. High-performance liquid chromatography-mass spectrometry identified 38 metabolites, including flavonoids, terpenoids, and phenols, which served as reducing and stabilizing agents. Optimized synthesis conditions included pH 9, an extract concentration of 10 mg mL^−1^, silver nitrate concentrations of 12 mM (leaf) and 10 mM (flower), and temperatures of 80 °C (leaf) and 70–80 °C (flower). AgNPs exhibited a uniform spherical shape, with mean diameters of 9.22 ± 1.97 nm (leaf-AgNPs) and 7.46 ± 1.58 nm (flower-AgNPs). Moreover, they demonstrated significant antibacterial activity against *Staphylococcus aureus* and *Escherichia coli*, with leaf-AgNPs showing superior efficacy (MIC: 16 μg mL^−1^) compared with flower-AgNPs (MIC: 32 μg mL^−1^). Furthermore, both types of AgNPs exhibited concentration-dependent cytotoxic effects against 4T1 and KYSE-150 cell lines through reactive oxygen species-mediated cytotoxicity, with leaf-AgNPs showing enhanced effectiveness. These findings demonstrate the potential of *X. sorbifolia*-derived AgNPs as promising candidates for biomedical applications, particularly as antimicrobial agents with potent cytotoxic activity against cancer cells.

## Abstract 2

Green-synthesized AgNPs from *Xanthoceras sorbifolia* leaf extract demonstrate superior antimicrobial and cytotoxic efficacy against cancer cells compared with flower-derived nanoparticles, suggesting enhanced biomedical application potential.

## Graphical abstract

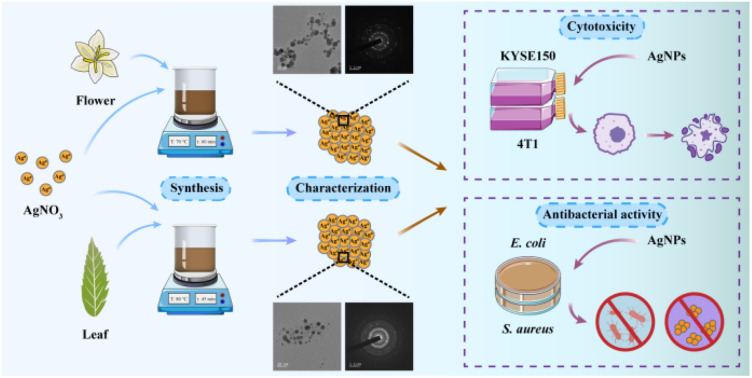

## 1. Introduction

Nanoparticles, which are fundamental components of nanomaterials, have garnered extensive attention for their application in diverse fields, such as sensing, catalysis, and environmental remediation.^1^ Among these, silver nanoparticles (AgNPs) stand out for their potent antibacterial and anticancer properties. AgNPs are considered essential broad-spectrum antibacterial agents in biomedicine, with applications in wound dressings, dental materials, and related fields.^2^ Moreover, AgNPs exhibit cytotoxicity against cancer cells through mechanisms such as the generation of reactive oxygen species (ROS) and modulation of gene expression, presenting significant potential in tumour treatment.^3,4^ The enhanced permeability and retention effect of tumours facilitates the selective accumulation of AgNPs in cancer cells while minimizing damage to healthy tissues.^5^ Beyond antibacterial and anticancer applications, AgNPs are employed in the medical field for anti-inflammatory therapy,^6^ diabetes management,^7^ and enhancing vaccine immunogenicity.^8^

The synthesis of AgNPs is generally achieved *via* three primary methods: physical, chemical, and biological. Physical methods are rapid and free from hazardous byproducts but are limited by low productivity and high energy requirements.^9^ Chemical synthesis offers consistent and tunable nanoparticle sizes but often involves toxic reagents, which make it less suitable for biomedical applications.^10^ In contrast, biological synthesis methods using plants, bacteria, fungi, or algae have emerged as safer and more eco-friendly alternatives.^11^ Among these, plant-based methods are particularly promising for large-scale AgNP production owing to easy tunability of their reaction conditions and their reliance on less toxic reagents.^12^

Plants contain a diverse array of biomolecules, such as terpenes, flavonoids, carboxylic acids, carbohydrates, proteins, and vitamins, which act as reducing and capping agents during AgNP synthesis.^13^ For example, AgNPs synthesized using *Hydrangea paniculata* flower extracts exhibit antibacterial and free-radical-scavenging capabilities,^14^ while those derived from *Thymus vulgaris* water extracts demonstrate antioxidant and anti-inflammatory properties.^15^ Likewise, a recent work has shown that AgNPs biofabricated from *Plectranthus barbatus* leaf extracts display significant free-radical-scavenging activity and exhibit interesting optical properties related to their semiconductor behaviour, underscoring the broad potential of plant-mediated synthesis.^16^ Compared to AgNPs synthesized *via* physical or chemical methods, plant-derived AgNPs exhibit lower toxicity, enhanced biological activity, and greater potential for biomedical applications.^17^

*Xanthoceras sorbifolia*, a lignified oil-producing species of the Sapindaceae family, is referred to as “Wen Guan Hua” in traditional Chinese medicine.^18^ Various bioactive compounds, including triterpenes, monoterpenes, flavonoids, and phenolic acids, have been identified in the trunk of *X. sorbifolia*, highlighting its potential in AgNP synthesis.^19^ Additionally, the active constituents of *X. sorbifolia* exhibit anti-inflammatory, antioxidant, and anti-tumour properties.^20–22^ However, to date, no published studies have explored the synthesis of AgNPs using extracts from *X. sorbifolia*.

This study reports the synthesis of AgNPs using extracts from the leaves and flowers of *X. sorbifolia* and the differences in the biological activities of AgNPs produced from these two plant parts. The research involved analysing the phytochemical composition of the leaf and flower extracts and employing these extracts for AgNPs synthesis. The synthesized nanoparticles were characterized using a range of techniques, including ultraviolet-visible (UV-vis) spectroscopy, transmission electron microscopy (TEM) coupled with energy-dispersive X-ray spectroscopy (EDS), X-ray diffraction (XRD), Fourier transform infrared spectroscopy (FTIR), X-ray photoelectron spectroscopy (XPS), and dynamic light scattering (DLS). Compared with conventional methods, this green synthesis approach is user-friendly, environmentally sustainable, and cost-effective, and yields promising results. Furthermore, the antibacterial activity of the synthesized AgNPs was evaluated against both Gram-positive and Gram-negative bacteria, while their cytotoxic effects were tested on tumour cells. The findings suggest the significant potential of *X. sorbifolia*-derived AgNPs in clinical applications in the biomedical field. Building upon the established novelty of utilizing *X. sorbifolia* for AgNPs synthesis, this study takes a significant step further. To the best of our knowledge, it provides the first direct evidence demonstrating that the choice between its leaf and flower extracts results in AgNPs with distinct physicochemical profiles. These differences, in turn, lead to significantly varied antimicrobial and cytotoxic activities.

## 2. Experimental details

### 2.1 Plant extract preparation

Fresh leaves and flowers of *X. sorbifolia* were collected from Jingyuan County, Gansu Province, China. The samples were authenticated by Dr Peiliang Liu from the College of Life Sciences, Northwest University, Xi'an, China, and voucher specimens were deposited at the Herbarium of the College of Life Sciences, Northwest University (WNU, No: 20230502021, 20230502022). The specimens were washed twice with ultra-pure water to remove dust and contaminants, followed by drying in an oven at 40 °C overnight. Based on prior findings, the highest concentration of phenolic components was achieved at 90 °C.^23^ Accordingly, 2 g of finely cut leaves and flowers were added to 100 mL of ultra-pure water and heated at 90 °C for 1 h. The extracts were filtered using Whatman No. 1 filter paper, sealed, and stored at 4 °C for subsequent nanoparticle synthesis.

### 2.2 Phytochemical analysis

Secondary plant metabolites are critical for the green synthesis of AgNPs.^24^ High-performance liquid chromatography coupled with mass spectrometry (HPLC-MS) was used to quantify the secondary metabolites present in the plant extracts. The plant samples were pulverized using a powder grinder, and 2.0 g of the ground material was placed in a clean conical flask, followed by the addition of 100 mL of ultra-pure water. The flask was incubated in a preheated water bath at 90 °C for 60 min. The resulting solution was filtered through Whatman No. 1 filter paper, and the filtrate was collected. One millilitre of the filtrate was diluted 10-fold, passed through a 0.22 μm membrane filter, and transferred to a liquid-phase sample vial. The samples were separated using an ACQUITY ultra-high-performance liquid chromatography system (Waters, MA, USA) equipped with an ACQUITY HSS C18 chromatographic column (2.1 × 100 mm, 1.8 μm). The mobile phase consisted of 0.1% formic acid in water (phase A) and pure methanol (phase B). The chromatographic eluate was analysed using an Agilent 5600 time-of-flight high-resolution mass spectrometer to obtain mass spectrometric data. The mass spectrometry conditions were as follows: negative ion mode, a source block temperature of 500 °C, a capillary voltage of 4500 V, a nebulizer gas pressure of 50 psi, and a collision energy of −10 eV. Mass-to-charge ratio (*m*/*z*) data were collected in the range of 50–1000 *m*/*z*.

### 2.3 Phytosynthesis of AgNPs

AgNPs were synthesized using plant extracts from *X. sorbifolia* as reducing agents. In the standard synthesis procedure, equal volumes of the plant extract and a silver nitrate (AgNO_3_) solution (5 mL each) were combined. The pH of the solution was adjusted using hydrochloric acid (HCl, 0.1 mM) and sodium hydroxide (NaOH, 0.1 mM). The mixture was stirred and heated to facilitate AgNP formation. After synthesis, the AgNP solution was centrifuged at 11 000 rpm for 30 min, and the pellet was washed twice with ethanol and dried under a vacuum to obtain dry AgNPs. The nanoparticles synthesized from the leaves and flowers of *X. sorbifolia* are referred to as leaf-AgNPs and flower-AgNPs, respectively, and were stored at 4 °C for subsequent analysis.

### 2.4 Optimization of reaction conditions for phytosynthesis

To optimize the yield of AgNPs synthesized from *X. sorbifolia* extracts, the effects of several reaction parameters were systematically investigated. These parameters included solution pH (5, 6, 7, 8, and 9), reaction time (15, 30, 45, 60, and 75 min), heating temperature (27, 60, 70, 80, and 90 °C), concentration of the plant extract (8, 10, 12, 14, and 16 mg mL^−1^), and concentration of AgNO_3_ (8, 10, 12, 14, and 16 mM). Table 1 outlines the tested variables and their corresponding constant reaction parameters. The absorption peaks of the synthesized products were analysed using UV-visible spectroscopy (Shimadzu UV-2500, Kyoto, Japan) to determine the optimal conditions for maximum AgNP production.

**Table 1:** Extraneous variables in the optimization of synthesis conditions

Parameter	Condition (leaf)	Condition (flower)	Variable
pH	Plant extract: 10 mg mL^−1^	Plant extract: 16 mg mL^−1^	6
	AgNO_3_: 12 mM	AgNO_3_: 10 mM	7
	Temperature: 80 °C	Temperature: 70 °C	8
	Time: 60 min	Time: 60 min	9
			10
Concentration of AgNO_3_ (mM)	pH: 9	pH: 9	8
	Plant extract: 10 mg mL^−1^	Plant extract: 16 mg mL^−1^	10
	Temperature: 80 °C	Temperature: 70 °C	12
	Time: 45 min	Time: 60 min	14
			16
Concentration of plant extract (mg mL^−1^)	pH: 9	pH: 9	16
	AgNO_3_: 12 mM	AgNO_3_: 10 mM	14
	Temperature: 80 °C	Temperature: 70 °C	12
	Time: 45 min	Time: 60 min	10
			8
Temperature	pH: 9	pH: 9	27
	Plant extract: 10 mg mL^−1^	Plant extract: 16 mg mL^−1^	60
	AgNO_3_: 12 mM	AgNO_3_: 10 mM	70
	Time: 45 min	Time: 60 min	80
			90
Reaction time	pH: 9	pH: 9	15
	Plant extract: 10 mg mL^−1^	Plant extract: 16 mg mL^−1^	30
	AgNO_3_: 12 mM	AgNO_3_: 10 mM	45
	Temperature: 80 °C	Temperature: 70 °C	60
			75

### 2.5 Characterization of green-synthesized AgNPs

The AgNPs synthesized using *X. sorbifolia* extracts were characterized using a variety of analytical techniques. The morphology and size of the nanoparticles were examined using TEM (JEM-F200, JEOL, Japan). EDS (JEM-F200, JEOL, Japan) was employed to determine the concentration of silver and other elemental components of the AgNPs. The crystalline structure of the nanoparticles was analysed by XRD (SmartLab SE, Rigaku, Japan). The functional groups present in the plant extracts and the synthesized AgNPs were identified using FTIR (TENSOR27, Bruker, Germany). XPS (PHI5000 VersaProbe III, ULVAC-PHI, Japan) was used to analyse the surface chemical composition of the nanoparticles and investigate the reduction of Ag^+^. Finally, the particle size distribution of the AgNPs was measured using DLS (Zetasizer Nano S90, Malvern, United Kingdom).

### 2.6 Antibacterial activity of AgNPs

The antibacterial efficacy of the synthesized AgNPs was evaluated against the Gram-positive bacterium *Staphylococcus aureus* (*S. aureus*, ATCC 25923) and the Gram-negative bacterium *Escherichia coli* (*E. coli*, ATCC 25922). A single bacterial colony was selected from solid Luria–Bertani (LB) medium and cultivated in LB liquid medium at 37 °C for 12 h. Subsequently, a portion of the bacterial culture was extracted and incubated at 37 °C for 3.5 h for activation. The turbidity of the bacterial suspension was adjusted to 0.5 McFarland standard prior to testing.

#### 2.6.1. Minimum inhibitory concentration (MIC) and minimum bactericidal concentration (MBC) assays

The broth dilution method was employed to determine the MIC and MBC values of leaf-AgNPs and flower-AgNPs against *S. aureus* and *E. coli*. These assays were conducted in triplicate, and the methodology was based on the guidelines provided by the Clinical and Laboratory Standards Institute (CLSI) for antimicrobial susceptibility testing, with necessary modifications for the assessment of nanoparticles. The AgNPs were diluted in sterile water to prepare a series of concentrations ranging from 4 to 64 μg mL^−1^. This concentration range was selected to establish a comprehensive dose–response curve for antibacterial efficacy, facilitating the determination of MIC and MBC values. Each diluted sample was mixed with a bacterial suspension containing 1 × 10^6^ CFU mL^−1^ in separate tubes. The negative control consisted of LB broth with bacterial strains but without AgNPs, while the positive control included 100 μg mL^−1^ of gentamicin and ampicillin.

After 24 h of incubation at 37 °C, optical density (OD) measurements were made at 600 nm (OD_600_) to determine the MIC, that is, the lowest concentration at which bacterial growth was completely inhibited. To determine the MBC, 100 μL of solution from each tube was plated onto nutrient agar and incubated at 37 °C for 24 h. The MBC was identified as the lowest concentration of AgNPs that eliminated visible bacterial growth on the agar plates.

#### 2.6.2. Growth curve measurement

The antibacterial kinetics of the synthesized AgNPs were investigated using a 96-well plate assay. Ninety microliters of a diluted bacterial suspension with a concentration of 1 × 10^6^ CFU mL^−1^ were added to each well of a sterile 96-well plate. Subsequently, 10 μL of the AgNP solution was added to achieve final concentrations of 1, 2, 4, 8, 16, 32, and 64 μg mL^−1^, with five replicates for each concentration. Blank and positive controls were included for each bacterial strain. The blank control consisted of 10 μL of LB liquid medium, while the positive control contained 10 μL of antibiotics at a concentration of 2 mg mL^−1^. OD_600_ values were recorded at the following time points: 2, 4, 6, 8, 10, 12, and 24 h. These measurements were used to assess bacterial growth inhibition over time and determine the antimicrobial kinetics of the AgNPs.

#### 2.6.3. Agar diffusion assay

The agar diffusion assay was performed to evaluate the antimicrobial properties of the AgNPs against both *S. aureus* and *E. coli*. A 5000-fold diluted bacterial suspension was adjusted to an OD_600_ value of 0.5 and evenly spread over solid LB medium. The plates were agitated for 1 min and allowed to stand upright for 5 min. Seven experimental groups were established for each bacterial strain, corresponding to AgNP concentrations ranging from 32 to 256 μg mL^−1^. For comparison, filter papers treated with a standard antibiotic, 1 mM AgNO_3_, and the plain plant extracts (leaf and flower) from *X. sorbifolia* were also included. Aseptic filter papers were placed on the agar surface, and 50 μL of the respective drug solution was applied to each paper. After the solutions were fully absorbed, the plates were inverted and incubated at 37 °C for 24 h. Following incubation, photographs were taken, and the diameter of the inhibition zones around each filter paper was measured to determine the antimicrobial activity of the AgNPs.

### 2.7 Cytotoxic properties of the AgNPs

#### 2.7.1. Cell cultures

Mouse breast cancer cells (4T1) and human esophageal cancer cells (KYSE-150) were obtained from Wuhan Pricella Biotechnology. The 4T1 cells were cultured in Roswell Park Memorial Institute (RPMI) 1640 medium (HyClone, USA) supplemented with 10% fetal bovine serum (FBS; Sijiqing Bioengineering, Hangzhou, China) and 1% penicillin–streptomycin (Beyotime Biotechnology, Shanghai, China). The KYSE-150 cells were maintained in a medium comprising 45% RPMI 1640, 45% Ham's F-12 Nutrient Mixture, 10% FBS, and 1% penicillin–streptomycin.

#### 2.7.2. CCK-8 assay

The Cell Counting Kit-8 (CCK-8) assay (Beyotime Biotechnology, Shanghai, China) was used to evaluate the cytotoxic effects of AgNPs on 4T1 and KYSE-150 cells. Briefly, 1 × 10^4^ cells were seeded into each well of a 96-well plate and incubated at 37 °C. Cells treated with only the culture medium served as the negative control. The cells were treated with various concentrations of AgNPs ranging from 1 to 64 μg mL^−1^ and incubated for 24 h. This concentration range was chosen based on preliminary experiments to ensure coverage of a full dose–response from minimal to maximal cytotoxic effects, facilitating the determination of IC50 values. Subsequently, the medium was replaced with fresh medium containing 5% CCK-8 and incubated for an additional 2 h. The absorbance of each well was measured at 450 nm using a multifunctional microplate reader (Synergy H1, BioTek, USA). Cell viability was expressed as percentage values relative to the untreated control group (set as 100%). Each experimental condition was tested in six replicates within a single experiment. The entire experiment was independently repeated at least three times (*n* ≥ 3).

#### 2.7.3. Measurement of ROS

To evaluate ROS generation, the 2,7-dichlorodihydrofluorescein diacetate (DCFDA) dye (Beyotime Biotechnology, Shanghai, China) was utilized. Both 4T1 and KYSE-150 cells were seeded in a 6-well plate at a density of 1 × 10^5^ cells per well. Untreated cells served as the negative control. The cells were then treated with AgNPs at concentrations ranging from 0 to 32 μg mL^−1^. After 12 h of incubation, the cells were stained with DCFDA diluted 1 : 1000 in the growth medium and incubated for 25 min. The fluorescent images were captured using a fluorescence microscope. For quantitative analysis, images from at least four different fields of view were analysed per condition within each independent experiment. The ROS fluorescence intensity was quantified using ImageJ software. This entire experimental procedure was independently repeated three times. The quantified intensity values were then normalized to the untreated control group (set as 100%) for comparative analysis.

### 2.8 Statistical analysis

All experiments were independently conducted at least three times, and the results are presented as mean ± standard deviation (SD). Statistical analysis was performed using one-way analysis of variance (ANOVA) followed by Tukey's multiple comparisons test in GraphPad Prism 8.0.0 software, with a significance threshold of *p* < 0.05. The AgNP characterization data were visualized and analysed using Origin 2021, ImageJ, and NanoMeasurer.

## 3. Results and discussion

### 3.1 Characterization of phytochemicals in *X. sorbifolia*

**Fig. 1:**
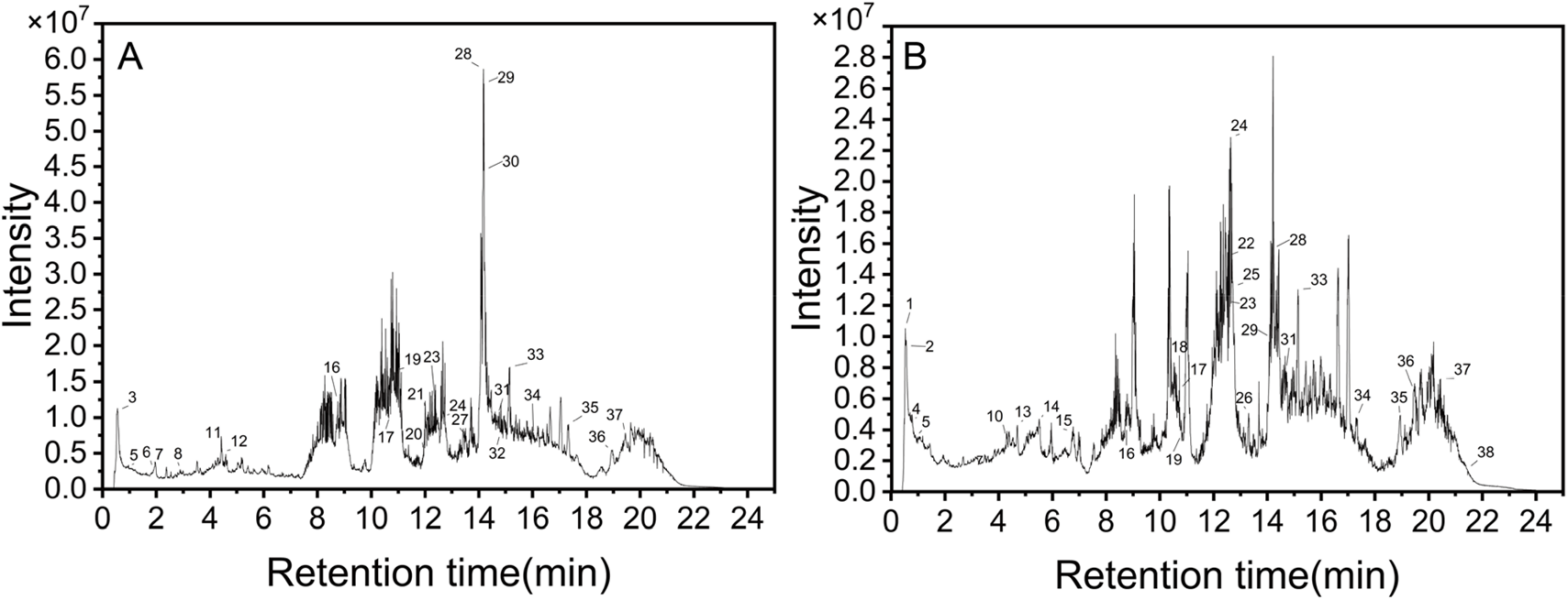
Total ion chromatogram of *X. sorbifolia* flowers (A) and leaves (B).

The HPLC-MS analysis of the leaf and flower extracts of *X. sorbifolia* identified a total of 38 primary metabolites, including flavonoids, glycosides, phenols, terpenoids, and other compounds (Fig. 1 and Table 2). Among these, flavonoids, such as myricitrin-3-*O*-rutinoside, myricetin, and quercitrin, were prominent due to their antioxidant properties, which operate through various mechanisms. These flavonoids also exhibited antibacterial, anti-inflammatory, and anti-cancer activities.^25–27^ Flavonoids derived from plant sources are particularly significant in the green synthesis of AgNPs as a direct correlation exists between the reducing capacity of plant extracts and their flavonoid concentration. This is attributed to the antioxidant properties of flavonoids, which facilitate the reduction of silver ions. Extracts rich in flavonoids have been observed to produce nanospheres with greater consistency during AgNP synthesis.^28^ Notably, the leaf extract of *X. sorbifolia* contained two catechin derivatives, namely epicatechin and gallocatechin, which were absent in the flower extract.

**Table 2:** Preliminarily identified metabolites in aqueous *X. sorbifolia* flower and leaf extracts

Retention time	Adduct/charge	Formula	Found at mass	Library hit	Source	Compound class
0.53	[M − H]^−^	C_7_H_12_O_6_	191.0559	d-(−)-Quinic acid	Flowers	Carboxylic acid
0.54	[M + Cl]^−^	C_12_H_22_O_11_	377.0843	Alpha-lactose	Leaves	Glycosylglucose
0.54	[M + Cl]^−^	C_12_H_22_O_11_	377.0843	Sucrose	Flowers	Carbohydrate
0.77	[M − H]^−^	C_11_H_17_NO_8_	290.0879	*N*-Fructosyl pyroglutamate	Flowers	Amino acid derivative
0.92	[M − H]^−^	C_13_H_16_O_10_	331.0659	Gallic acid-4-*O*-β-d-glucoside	Leaves and flowers	Flavonoid
1.89	[M − H]^−^	C_9_H_11_NO_2_	164.0718	l-Phenylalanine	Leaves	Amino acid
1.98	[2M − H]^−^	C_15_H_14_O_7_	611.1376	(−)-Gallocatechin	Leaves	Alcohol
2.91	[M − H]^−^	C_15_H_14_O_6_	289.0719	Epicatechin	Leaves	Flavonoid
4.3	[M − H]^−^	C_20_H_30_O_8_	443.1908	Ptaquiloside	Flowers	Terpene
4.42	[2M − H]^−^	C_15_H_18_O_8_	651.1921	Bilobalide	Leaves	Terpene
4.42	[M − H]^−^	C_9_H_8_O_3_	163.0404	4-Coumarate	Leaves	Carboxylic acid
4.69	[M + FA–H]^−^	C_15_H_24_O_9_	393.1754	Leonuride	Flowers	Glycoside
5.51	[M − H]^−^	C_19_H_28_O_11_	431.1911	Osmanthuside H	Flowers	Glycoside
6.74	[M − H_2_O–H]^−^	C_17_H_24_O_10_	433.2063	Verbenalin	Flowers	Glycoside
8.75	[M − H]^−^	C_21_H_20_O_13_	479.0819	Myricetin 3-*O*-β-d-galactopyranoside	Leaves and flowers	Flavonoid
10.56	[M − H]^−^	C_27_H_30_O_16_	609.1438	Quercetin-3-*O*-rutinoside	Leaves	Flavonoid
10.71	[2M − H]^−^	C_21_H_20_O_12_	927.18	Hyperin	Leaves and flowers	Flavonoid
10.73	[M + FA–H]^−^	C_29_H_50_O_2_	475.1803	Alpha-tocopherol	Flowers	Tocopherol
10.83	[M − H]^−^	C_21_H_20_O_12_	463.0869	Myricitrin	Leaves and flowers	Flavonoid
11.93	[M − H]^−^	C_21_H_20_O_12_	463.0868	Quercetin-3-*O*-galactoside	Leaves and flowers	Flavonoid
12.02	[M − H]^−^	C_32_H_38_O_21_	771.1975	Isoorientin 3,6-di-*O*-glucoside	Leaves	Carbohydrate derivative
12.46	[M − H]^−^	C_21_H_20_O_12_	463.0872	Isoquercitrin	Flowers	Flavonoid
12.46	[M − H]^−^	C_33_H_40_O_19_	739.2057	Kaempferol 3-*O*-(2,6-di-*O*-alpha-l-rhamnopyranosyl)-beta-d-galactopyranoside	Leaves and flowers	Flavonoid
12.63	[M − H]^−^	C_27_H_30_O_16_	609.1444	Aureusidin 4,6-diglucoside	Leaves and flowers	Phenol
12.71	[M − H]^−^	C_27_H_30_O_15_	593.1496	Kaempferol-7-*O*-neohesperidoside	Flowers	Flavonoid
13.3	[M − H]^−^	C_27_H_30_O_15_	593.1492	Lonicerin	Flowers	Flavonoid
13.53	[M − H]^−^	C_15_H_10_O_8_	317.0289	Myricetin	Leaves	Flavonoid
14.17	[M − H]^−^	C_21_H_22_O_11_	449.0968	Astilbin	Leaves and flowers	Flavonoid
14.18	[2M − H]^−^	C_21_H_36_O_10_	895.1921	Kenposide A	Leaves	Glycoside
14.18	[M − H]^−^	C_21_H_20_O_11_	447.0925	Quercitrin	Leaves and flowers	Flavonoid
14.69	[M + FA–H]^−^	C_28_H_32_O_16_	623.1587	Isorhamnetin-3-*O*-rutinoside	Leaves and flowers	Flavonoid
14.8	[M + Br]^−^	C_31_H_48_O_7_	531.2112	Phytolaccagenin	Leaves	Terpenoid
15.14	[M − H]^−^	C_21_H_20_O_10_	431.0984	Afzelin	Leaves and flowers	Flavonoid
16.04	[M − H]^−^	C_15_H_10_O_6_	285.0394	Kaempferol	Leaves	Flavonoid
17.33	[M − H]^−^	C_18_H_32_O_5_	327.2162	(10*E*,15*Z*)-9,12,13-Trihydroxy-10,15-octadecadienoic acid	Leaves and flowers	Fatty acid
18.94	[M − H]^−^	C_18_H_34_O_5_	329.2328	Pinellic acid	Leaves and flowers	Fatty acid
19.46	[M − H]^−^	C_16_H_32_O_4_	287.2231	10,16-Dihydroxyhexadecanoic acid	Leaves and flowers	Fatty acid
21.43	[M − H]^−^	C_27_H_30_O_14_	577.2671	Chrysin 7-gentiobioside	Flowers	Glycoside

Terpenoids, another prominent class of metabolites, are known to adsorb onto metal nanoparticles at higher concentrations. This interaction is hypothesized to occur through π-electron interactions or carbonyl group binding in the absence of stronger ligating agents.^29^ For instance, Ali *et al.* demonstrated the role of terpenoids in the synthesis of copper oxide nanoparticles.^30^ Phenolic compounds, such as aureusidin 4,6-diglucoside, were detected in both leaf and flower extracts. These phenolic compounds serve dual roles as reducing agents and stabilizers during the synthesis of AgNPs and hence, play an important role in nanoparticle production.^31^ Additionally, carbohydrates (glucosides) and their derivatives were identified in the extracts. These compounds are believed to play a critical role in reducing silver ions and preventing AgNP agglomeration.^32^ A notable glucoside, kenposide A, has been identified as a reducing agent that facilitates AgNP formation. It also exhibits biological activity by inhibiting 5-lipoxygenase, a key enzyme involved in leukotriene synthesis.^32^ Verbenalin, another compound detected in the extracts, has shown efficacy in reducing Aβ trophin generation in *in vitro* cell assays and animal models of Alzheimer's disease.^33^

Three fatty acids, including (10*E*,15*Z*)-9,12,13-trihydroxy-10,15-octadecadienoic acid, pinellic acid, and 10,16-dihydroxyhexadecanoic acid, were also identified in the extracts. Previous studies have reported a high abundance of fatty acids in the fruit of *X. sorbifolia*, suggesting their potential as stabilizers in nanoparticle synthesis.^34^ Furthermore, d-(−)-quinic acid, a carboxylic acid found in the flower extract, is known to influence hemostasis significantly,^35^ thereby adding to the potential biological applications of the green-synthesized AgNPs. These findings highlight the rich phytochemical composition of *X. sorbifolia* extracts and their crucial roles in the reduction, stabilization, and functional enhancement of AgNPs, demonstrating that they are ideal candidates for green nanoparticle synthesis and biomedical applications.

### 3.2 Synthesis of AgNPs

**Fig. 2:**
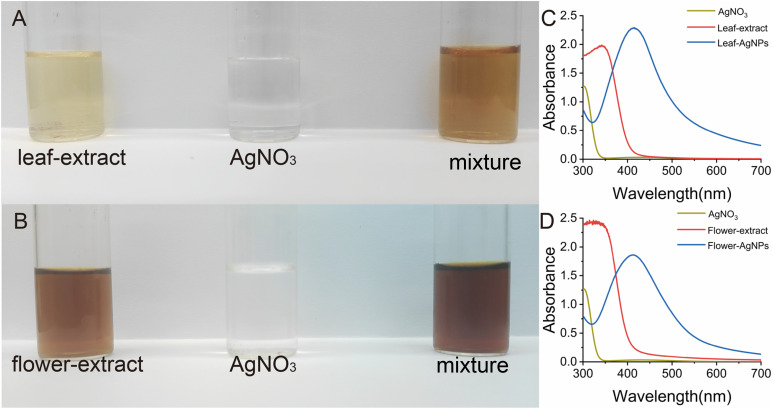
(A and B) Colour variations during the synthesis of AgNPs, and (C and D) the corresponding UV-visible spectra.

The successful synthesis of AgNPs was evident when plant extracts were combined with the AgNO_3_ solution. When examined separately, the plant extracts and AgNO_3_ solution remained light yellow or colourless, showing no indication of nanoparticle formation. However, upon mixing the plant extract with AgNO_3_, the solution turned brown (Fig. 2), signifying the reduction of silver ions to elemental silver, a process facilitated by the capping and reducing agents present in the plant extracts.^14^ The UV-visible spectrophotometric analysis further confirmed the synthesis of AgNPs. Neither the AgNO_3_ solution nor the plant extract alone exhibited distinct absorption peaks. In contrast, the mixture of plant extracts and AgNO_3_ displayed a significant absorption peak in the 350–450 nm range, which is characteristic of the surface plasmon resonance (SPR) phenomenon of AgNPs.^36^ The intensity of the SPR absorption peak increased proportionally with the concentration of AgNPs, indicating successful nanoparticle synthesis. A sharp and narrow peak suggested a high degree of dispersion and uniformity of AgNPs in the solution. Furthermore, a blue shift (towards shorter wavelengths) of the maximum absorption peak was observed, indicating a reduction in the size of the synthesized AgNPs.^37^ These findings demonstrate the critical role of plant extracts in reducing silver ions and stabilizing the resultant AgNPs, and the SPR phenomenon provided a reliable optical signature for monitoring nanoparticle synthesis and dispersion quality.

### 3.3 Optimization of the reaction conditions of AgNP synthesis

#### 3.3.1. Effect of pH

**Fig. 3:**
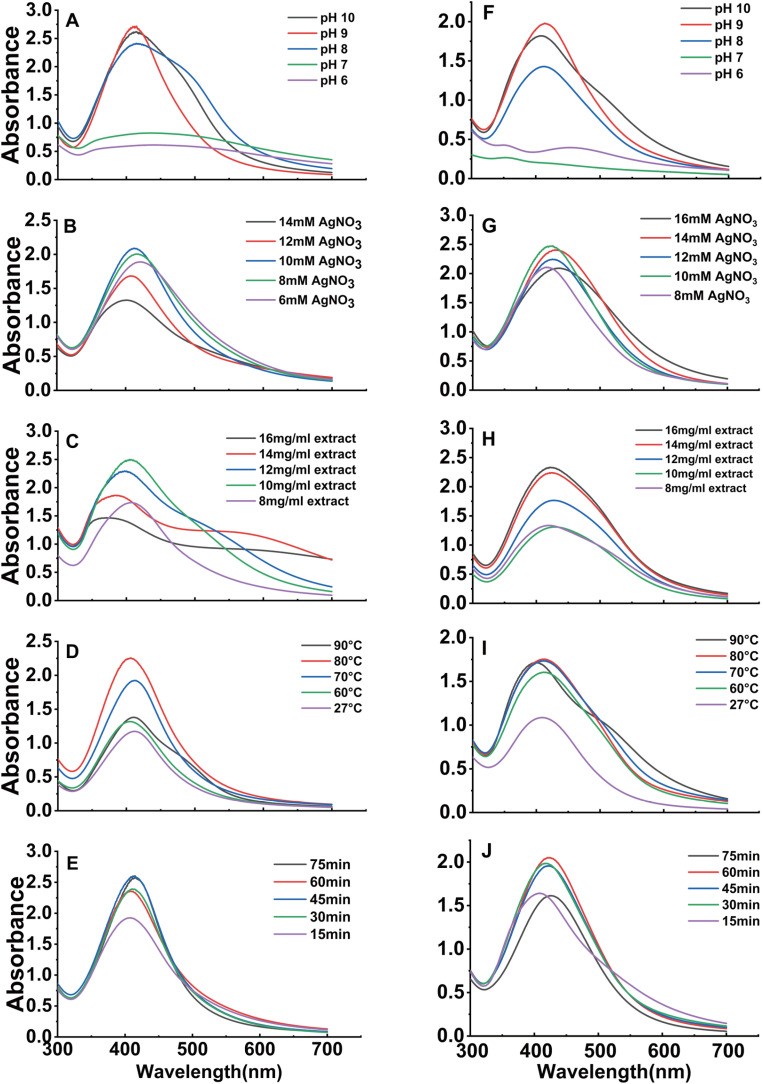
The impact of pH, AgNO_3_ concentration, plant extract concentration, temperature, and reaction time on the production of AgNPs using aqueous extracts of *X. sorbifolia* leaves (A–E) and *X. sorbifolia* flowers (F–J).

The formation of AgNPs was significantly influenced by the pH of the reaction medium. The synthesis process can be divided into two stages. Initially, the silver ions react with alkaline ions to form silver oxide (Ag_2_O), which is subsequently reduced to colloidal silver by a reducing agent. In the second stage, colloidal silver reacts further with silver ions, enabling continuous reduction. The addition of NaOH to the system enhances the surface area of the silver nuclei, thereby accelerating the reaction rate.^38^ Flavonoids present in the leaves and flowers of *X. sorbifolia* exhibited strong reducing properties in an alkaline environment, facilitating rapid nucleation and particle formation. However, these reducing properties were significantly diminished in acidic or neutral environments.^39^ As shown in Fig. 3A and F, the maximum absorption peaks for both leaf and flower extracts were observed at a pH of 9, with the leaf extract exhibiting a sharper peak. This difference may be attributed to variations in the distribution of reducing agents within the plant material. At pH 10, the intensity of the SPR peaks decreased for both extracts, likely because of the formation of larger AgNPs and reduction in the reaction rate at higher pH.^40^

#### 3.3.2. Effect of AgNO_3_ concentration

Fig. 3B and G illustrate the effects of varying AgNO_3_ concentrations on AgNP synthesis. For the leaf extract of *X. sorbifolia*, the highest and sharpest SPR peak was observed at a concentration of 12 mM. In contrast, the flower extracts exhibited an optimal absorption peak at 10 mM, indicating this was the most favourable concentration for nanoparticle synthesis. As the concentration of AgNO_3_ increased beyond these optimal levels, the SPR peaks broadened, and the absorption intensity decreased. This trend suggests an increase in the size of the synthesized AgNPs and a reduction in yield due to agglomeration.

#### 3.3.3. Effect of extract concentration

An increase in the concentration of the extract solution derived from *X. sorbifolia* flowers led to a corresponding elevation in the absorption peak, as shown in Fig. 3C and H. This observation aligns with previous experimental findings.^41^ For the leaf extracts, the absorption peak reached its maximum at a concentration of 10 mg mL^−1^ (Fig. 3C). However, any further increase in the concentration of the plant extract resulted in a decline in the absorption peak. This trend may be attributed to the enhanced bridging effect between the framework nanoparticles at higher concentrations of the reducing agent, potentially causing aggregation and a subsequent reduction in the reaction rate.^42^ It is important to note that these optimal concentrations (10 mg mL^−1^ for leaf and 16 mg mL^−1^ for flower) were established *via* systematic optimization. The goal of this optimization was to obtain the highest yield and best quality AgNPs for each plant part. This approach ensures that our comparative biological activity assessment reflects the full potential of the AgNPs synthesized under the most favorable conditions.

#### 3.3.4. Effect of reaction temperature

Fig. 3D and I illustrate the effect of temperature on AgNP production. With the leaf extract, the highest yield of AgNPs was achieved at 80 °C. Beyond this temperature, the yield decreased, likely due to excessive heat disrupting the reaction process. In contrast, the flower extracts exhibited a slight increase in yield when the temperature exceeded 70 °C. However, a significant decline was observed at 90 °C. At elevated temperatures, most silver ions are consumed during the nucleation process, consequently impeding the secondary reduction process on the surface of the nuclei.^43^ Cínthia C. Bonatto *et al.* suggested that increasing the reaction temperature can lead to the formation of AgNPs with smaller diameters.^44^ Interestingly, both leaf and flower extracts also exhibited distinct SPR absorption peaks at room temperature, indicating their ability to synthesize AgNPs under ambient conditions. This observation is consistent with previous studies showing that AgNPs can be synthesized using various plant sources at room temperature. However, this process requires a longer reaction duration than synthesis at elevated temperatures.^45,46^

#### 3.3.5. Effect of reaction time

The effect of reaction time on the synthesis of AgNPs was evaluated by varying the incubation duration. As shown in Fig. 3E and J, the production of leaf-AgNPs reached its maximum after 45 min of incubation, whereas flower-AgNPs exhibited peak production at 60 min. The synthesis of AgNPs was observed to increase with the reaction time, up to the respective optimal duration for each extract. Beyond these time points, no significant increase in nanoparticle production was observed.

In summary, the optimal reaction conditions for the synthesis of AgNPs were determined as follows: a pH of 9, an AgNO_3_ concentration of 12 mM for leaf extracts and 10 mM for flower extracts, an extract concentration of 10 mg mL^−1^, and a temperature of 80 °C for leaf extracts and 70–80 °C for flower extracts. The optimal incubation time was identified as 45 min for leaf-AgNPs and 60 min for flower-AgNPs. These parameters ensured efficient reduction, uniform particle formation, consistent nanoparticle size, and high yield. AgNPs synthesized under these optimal reaction conditions were utilized in all subsequent experiments.

### 3.4 Characterization of AgNPs

#### 3.4.1. XRD analysis

**Fig. 4:**
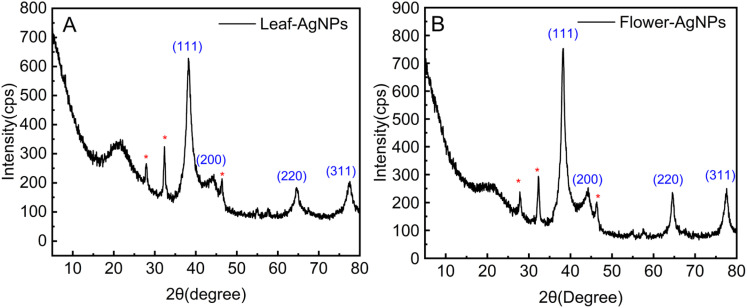
XRD patterns of AgNPs synthesized from *X. sorbifolia* leaves (A) and flowers (B).

The crystal structures of the AgNPs synthesized from the leaf and flower extracts of *X. sorbifolia* were analysed using XRD (Fig. 4). The XRD spectrum of the flower-AgNPs revealed four prominent peaks at 38.31°, 44.25°, 64.66°, and 77.6°, corresponding to the characteristic diffraction peaks of silver at the (111), (200), (220), and (311) crystal planes, respectively. Similarly, the leaf-AgNPs displayed four distinct peaks at 38.27°, 44.51°, 64.67°, and 77.74°, aligning with the same crystal planes of silver. The XRD results confirm the crystalline nature of the synthesized AgNPs produced using the leaf and flower extracts, with the face-centered cubic (FCC) structure characteristic of silver. In addition to the characteristic peaks of silver, unassigned peaks (indicated by *) were observed in the spectra of both leaf-AgNPs and flower-AgNPs. These peaks may correspond to the organic compounds present in the plant extracts, which are known to adhere to the surface of the AgNPs during crystallization, as reported previously.^47^ The presence of unassigned peaks also highlights the role of phytochemicals found in plant extracts in stabilizing the nanoparticles.

#### 3.4.2. FTIR analysis

**Fig. 5:**
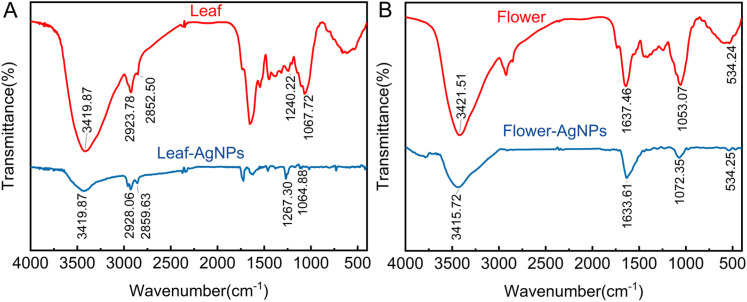
FTIR spectra of *X. sorbifolia* leaf (A) and flower (B) extracts and their derived AgNPs.

The FTIR spectra revealed several characteristic absorption peaks, indicative of functional groups involved in the synthesis and stabilization of AgNPs (Fig. 5). A broad absorption peak around 3420 cm^−1^ was observed and could be attributed to the hydroxyl (–OH) stretching vibrations of carboxylic acids and phenols, as well as N–H stretching vibrations.^48^ This indicates the involvement of hydroxyl-containing compounds in the reduction and capping of AgNPs. Additionally, a peak was observed at 2915 cm^−1^ in the spectra of both the plant extracts and the synthesized AgNPs, corresponding to the vibrations of aliphatic hydrocarbons containing methylene (–CH_2_) groups. A prominent absorption peak at 1633 cm^−1^ was observed in the spectra of both the *X. sorbifolia* extracts and the AgNPs. This peak is associated with amide bending vibrations,^49^ suggesting the presence of proteins in the plant extracts and their subsequent attachment to the AgNPs.^50^ The interaction of proteins with AgNPs likely plays a role in their stabilization. Furthermore, an absorption band at around 1373 cm^−1^, which is ascribed to the aromatic ring structures of polyphenolic compounds, was identified in both the leaf extracts and synthesized AgNPs.^51^ Polyphenols are known to act as effective reducing and stabilizing agents during nanoparticle synthesis. Another significant peak was observed at approximately 1060 cm^−1^, corresponding to the stretching of the C–O bonds in alcohols and ether compounds,^52^ further confirming the involvement of plant metabolites in capping the AgNPs.

The FTIR spectrum demonstrated a strong resemblance between the plant extracts and the synthesized AgNPs, indicating that multiple chemical constituents from the plant had adhered to the surface of the nanoparticles. These findings suggest that green-synthesized AgNPs may exhibit enhanced biological activity compared with those synthesized through conventional methods. Moreover, the functional groups identified in the FTIR analysis align closely with the phytochemicals detected in the HPLC-MS analysis, including phenols, flavonoids, and proteins, which are implicated in the reduction, stabilization, and capping processes during AgNP synthesis.

#### 3.4.3. TEM coupled with EDS analysis

**Fig. 6:**
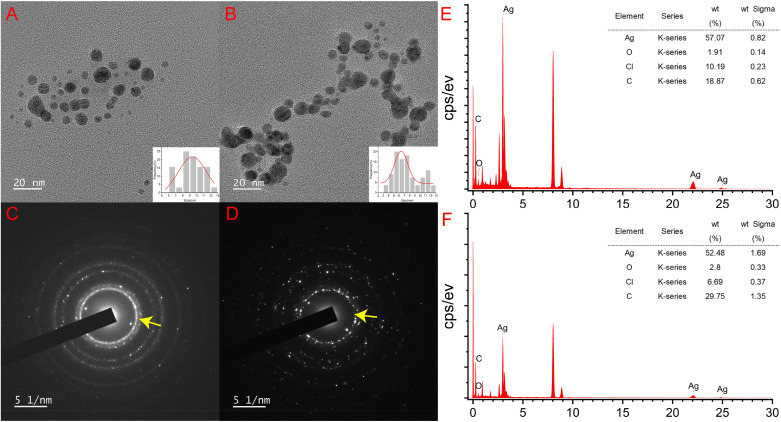
TEM analysis of AgNPs. TEM images of leaf-AgNPs (A) and flower-AgNPs (B); scale bar: 20 nm. The SAED patterns of leaf-AgNPs (C) and flower-AgNPs (D). EDX analysis of leaf-AgNPs (E) and flower-AgNPs (F). Yellow arrows: (111) crystal plane of AgNPs.

The TEM analysis revealed that the colloidal AgNPs synthesized *in situ* exhibited a high degree of dispersion, with a majority displaying a uniform spherical shape (Fig. 6A and B). The size distribution of the AgNPs ranged from 2 to 13 nm, and the predominant size range was 5 to 8 nm. The mean diameter of leaf-AgNPs was calculated to be approximately 9.22 ± 1.97 nm, while the mean diameter of flower-AgNPs was 7.46 ± 1.58 nm. These findings are consistent with the results of previous studies, confirming the successful synthesis of uniformly distributed AgNPs.^53^Fig. 6C and D illustrate the electron diffraction patterns of the AgNPs. The yellow arrows indicate positions corresponding to the (111) crystal plane of the AgNPs, which corroborate the XRD results. This alignment provides further evidence of the crystalline structure of the synthesized AgNPs, confirming their successful formation.

The EDS analysis, as presented in Fig. 6E and F, provided further insight into the elemental composition of the synthesized AgNPs. Distinct silver peaks were observed, accounting for 57.07% and 52.48% of the elemental composition of leaf-AgNPs and flower-AgNPs, respectively. Additionally, peaks corresponding to carbon and oxygen were also detected, indicating the presence of organic compounds from the plant extracts. The higher silver content in leaf-AgNPs suggests a more efficient reduction process than the flower-AgNPs. Conversely, the greater presence of non-silver components in flower-AgNPs is likely due to the higher concentration of flower extract (16 mg mL^−1^) used during the synthesis process, compared with 10 mg mL^−1^ of the leaf extract. The non-silver peaks, primarily carbon and oxygen, indicate that the phytochemicals in the plant extracts actively participated in the reduction and stabilization processes. These compounds adhered to the surface of the AgNPs, acting as capping agents and contributing to their stability.^54^

#### 3.4.4. XPS analysis

**Fig. 7:**
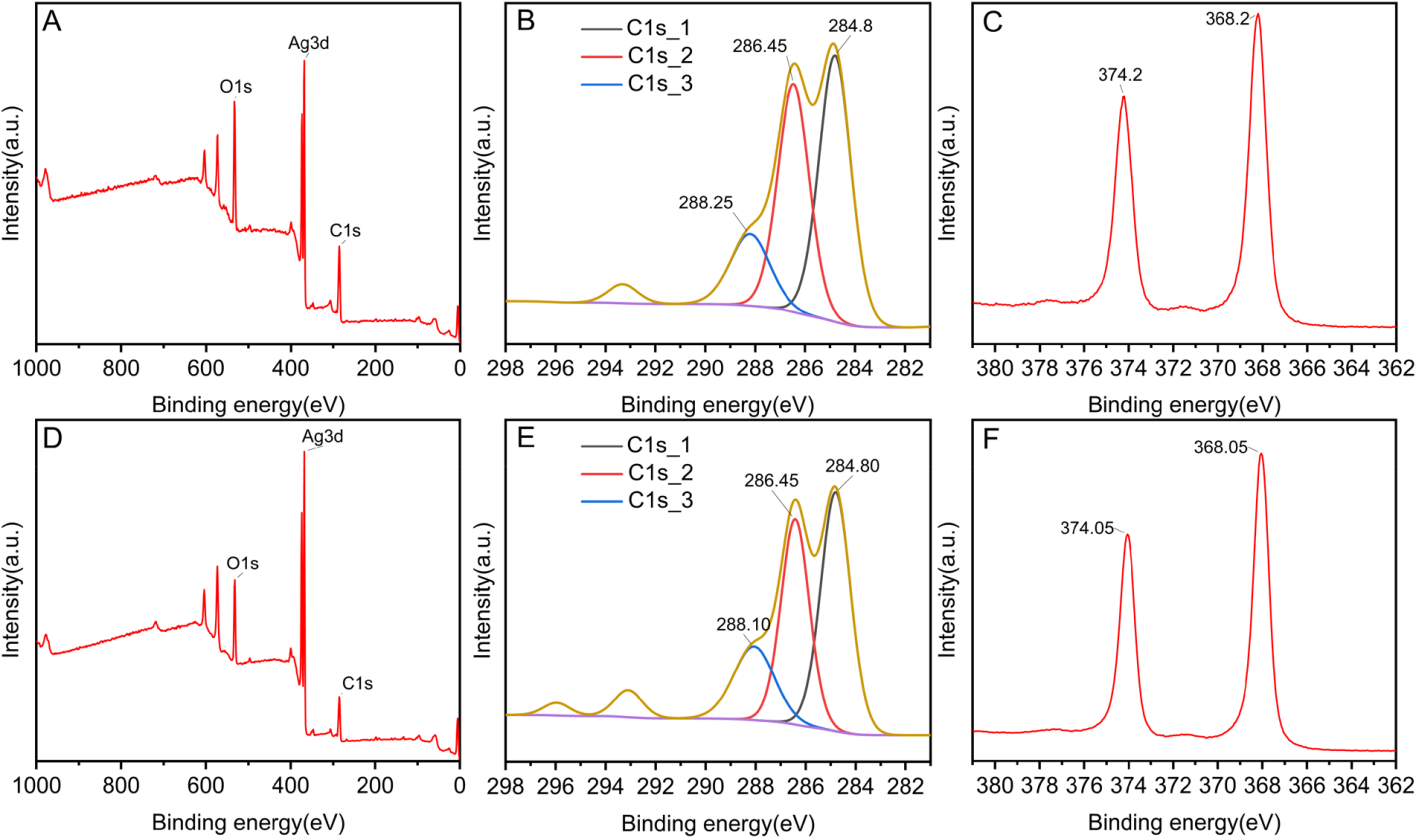
XPS spectra of leaf-AgNPs (A–C) and flower-AgNPs (D–F): survey (A and D); Ag 3d (B and E); C 1s (C and F).

The surface elemental composition and chemical states of the synthesized AgNPs were examined using XPS, as shown in Fig. 7. The wide survey scans of leaf-AgNPs (Fig. 7A) and flower-AgNPs (Fig. 7D) revealed the presence of three primary elements on their surfaces: carbon (C), silver (Ag), and oxygen (O). Among them, the peak corresponding to Ag was the most prominent, while the concentration of oxygen exceeded that of carbon, which is in agreement with the EDS findings. Fig. 7B and E highlight the surface carbon signals of the AgNPs. The spectra displayed three distinct peaks; the peak at 284.8 eV was attributed to C–C or C=C bonds. The peak at 286.45 eV corresponded to the C–O bonds, and the peak at 288 eV indicated the presence of O–C=O bonds.^55,56^ These findings suggest the plant-derived organic compounds adhered to the AgNPs surface and likely contributed to the reduction and stabilization processes during synthesis. Fig. 7C and F present the high-resolution XPS results of the Ag 3d region. For leaf-AgNPs, the Ag 3d_5/2_ absorption peak was observed at 374.2 eV, while the Ag 3d_3/2_ peak was located at 368.2 eV. Similarly, for flower-AgNPs, the Ag 3d_5/2_ and Ag 3d_3/2_ peaks were found at 374.05 eV and 368.05 eV, respectively. The energy separation between the 3d_5/2_ and 3d_3/2_ peaks was measured to be 6 eV for both types of AgNPs, confirming the successful reduction of Ag^+^ to Ag^0^.^57^

#### 3.4.5. DLS analysis

**Fig. 8:**
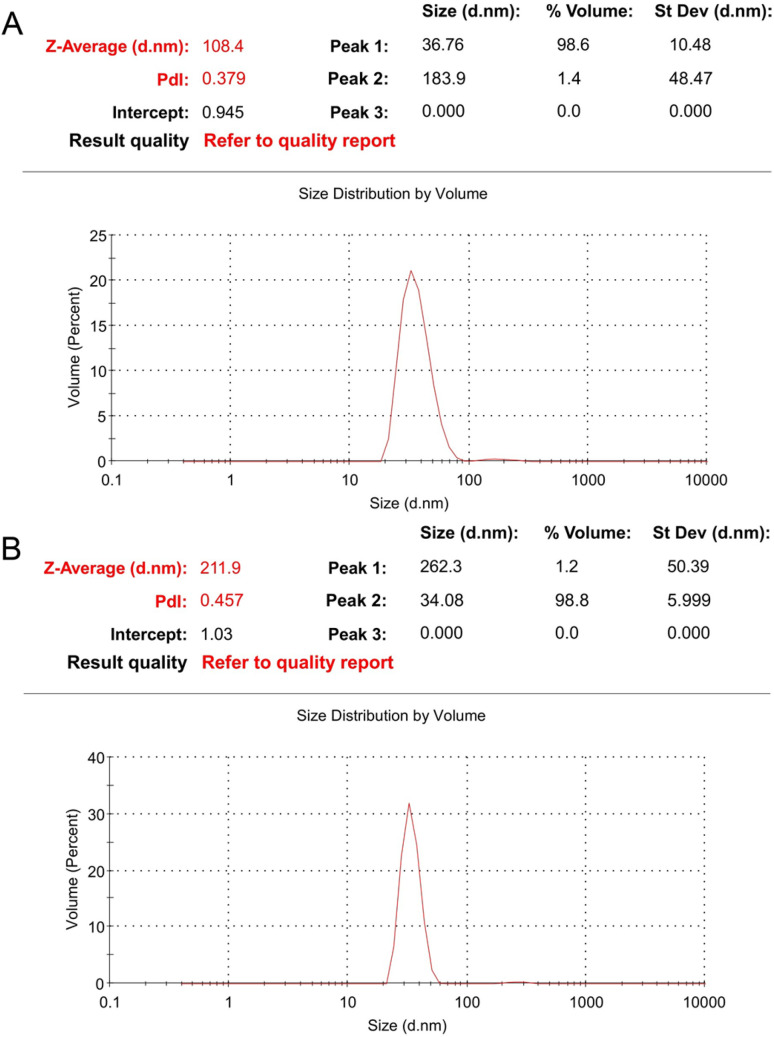
Size distribution of leaf-AgNPs (A) and flower-AgNPs (B).

A DLS analysis was performed to evaluate the particle size distribution of the synthesized AgNPs (Fig. 8). The particle sizes of the AgNPs ranged from 20 to 100 nm. The mean hydrodynamic radius of the leaf-AgNPs was determined to be 38.60 nm, while the mean hydrodynamic radius of the flower-AgNPs was calculated as 36.82 nm. Notably, these results are inconsistent with the particle size distribution observed in the TEM analysis. This discrepancy may be attributed to the differences in sample preparation. DLS measures the hydrodynamic diameter of nanoparticles in solution, which includes the plant-derived capping agents surrounding the nanoparticles.^58^ In contrast, the TEM samples were analysed after a drying and reconstitution process, which likely reduced the contribution of capping agents to the observed particle size. It is a common observation that DLS measurements yield larger sizes for green-synthesized nanoparticles compared with TEM due to the hydration layer and adsorbed biomolecules. However, this discrepancy does not invalidate the comparative bioactivity results. The synthesis and testing of both AgNPs types under comparable conditions ensure that their relative efficacy can be meaningfully assessed.

### 3.5 Antibacterial activity of AgNPs

#### 3.5.1. MIC and MBC assay

**Fig. 9:**
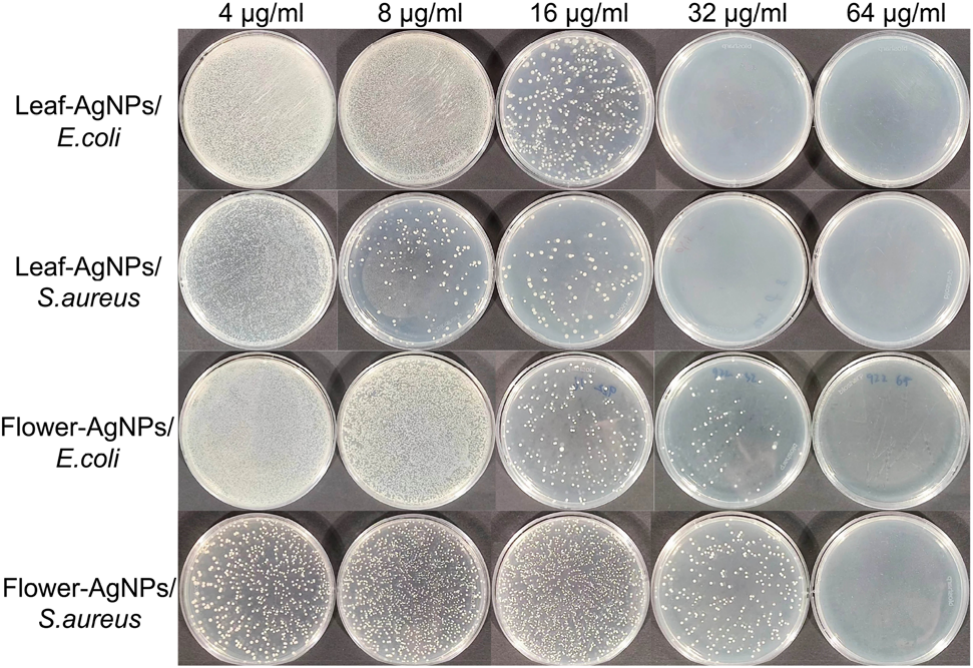
Bacterial growth on plates at different AgNP concentrations.

Previous studies have demonstrated variability in the antibacterial activity of AgNPs against these species, which can be attributed to differences in cell membrane thickness.^59^ The MIC and MBC values of the leaf-AgNPs and flower-AgNPs against the bacteria are depicted in Fig. 9. For leaf-AgNPs, the MIC was 16 μg mL^−1^ against both *S. aureus* and *E. coli*, while the MBC was 32 μg mL^−1^ for both strains. In contrast, the MIC of flower-AgNPs was 32 μg mL^−1^, with an MBC of 64 μg mL^−1^ for both bacteria. These results indicate that the leaf-AgNPs exhibited superior antibacterial activity to flower-AgNPs.

The enhanced antibacterial properties of plant-synthesized AgNPs compared to conventionally synthesized AgNPs have been attributed to the presence of bioactive compounds, such as flavonoids, phenols, and polyphenols, on their surfaces.^60^ The observed differences in antibacterial efficacy between leaf-AgNPs and flower-AgNPs may be linked to the unique phytochemical compositions of the respective plant extracts. The HPLC-MS analysis revealed that the leaf extract of *X. sorbifolia* contained two catechin derivatives, epicatechin and gallocatechin, which were absent in the flower extract. Prior studies have demonstrated a relationship between the lipophilicity of catechin derivatives and their ability to disrupt bacterial membranes, as well as their effectiveness against bacterial infections.^61^ These derivatives likely exert antibacterial effects by adsorbing to bacterial membranes and disrupting membrane functions.

#### 3.5.2. Antibacterial kinetics

**Fig. 10:**
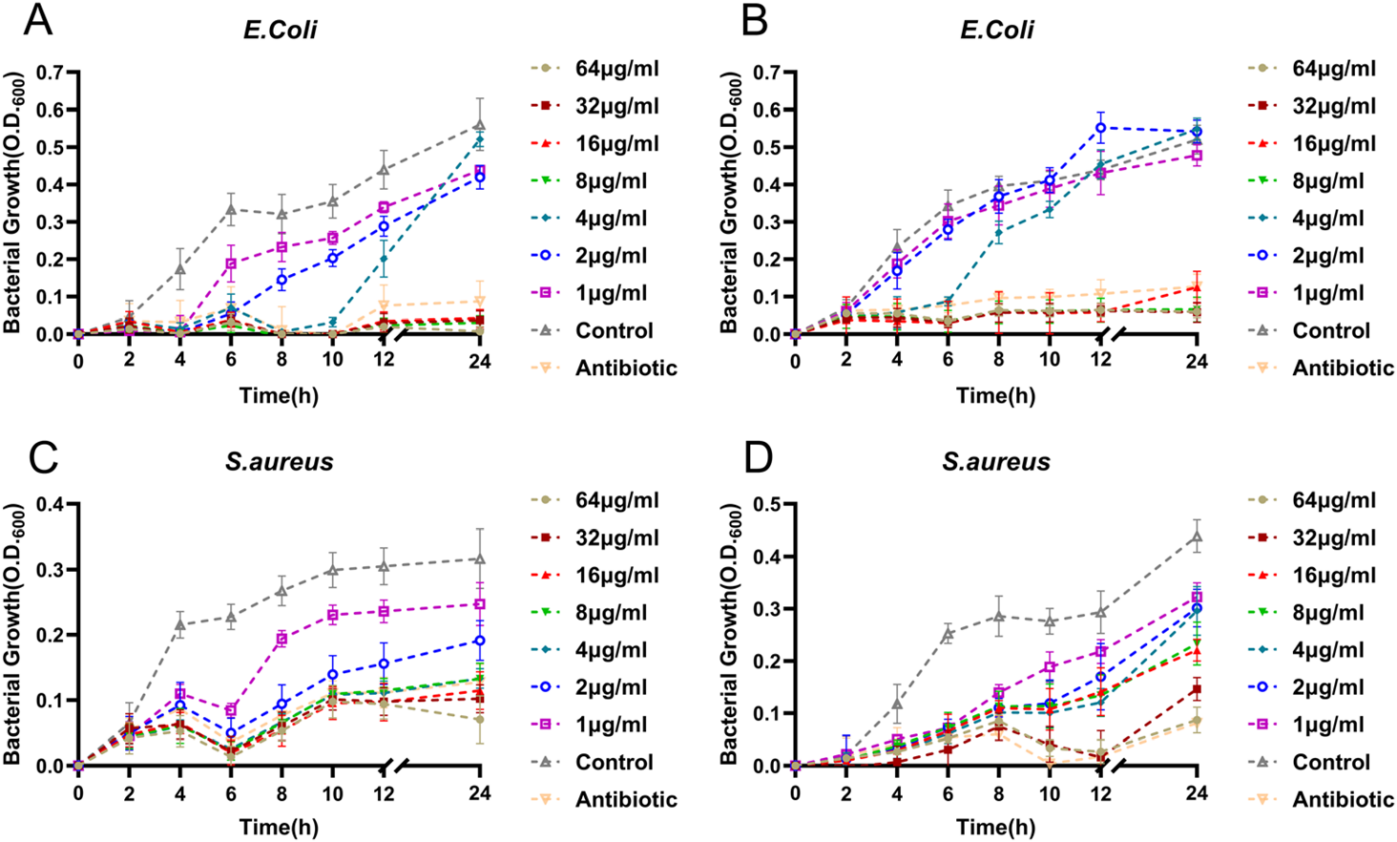
Effects of different concentrations of AgNPs on bacterial growth dynamics. (A) and (C) Leaf-AgNPs, (B) and (D) flower-AgNPs.

To further evaluate the antibacterial kinetics of the synthesized AgNPs, the OD_600_ values of *E. coli* and *S. aureus* cultures were measured at various time intervals (Fig. 10). The antibacterial activity of leaf-AgNPs against both bacterial strains was comparable to that of gentamicin at a concentration of 8 μg mL^−1^. In contrast, the flower-AgNPs exhibited significant antibacterial activity against *E. coli* at a concentration of 8 mg mL^−1^, whereas a higher concentration of 32 mg mL^−1^ was required to achieve a similar effect against *S. aureus*. These results suggest that the leaf-AgNPs are more effective than flower-AgNPs, likely due to the presence of unique bioactive compounds in the leaf extract, such as catechin derivatives, which may enhance their antibacterial activity. Additionally, this difference in efficacy may be attributed to the structural characteristics of the bacterial cell membrane. Gram-positive bacteria like *S. aureus* have a thicker peptidoglycan layer, which may act as a barrier to AgNP penetration and reduce their effectiveness.

#### 3.5.3. Disk diffusion assay

**Fig. 11:**
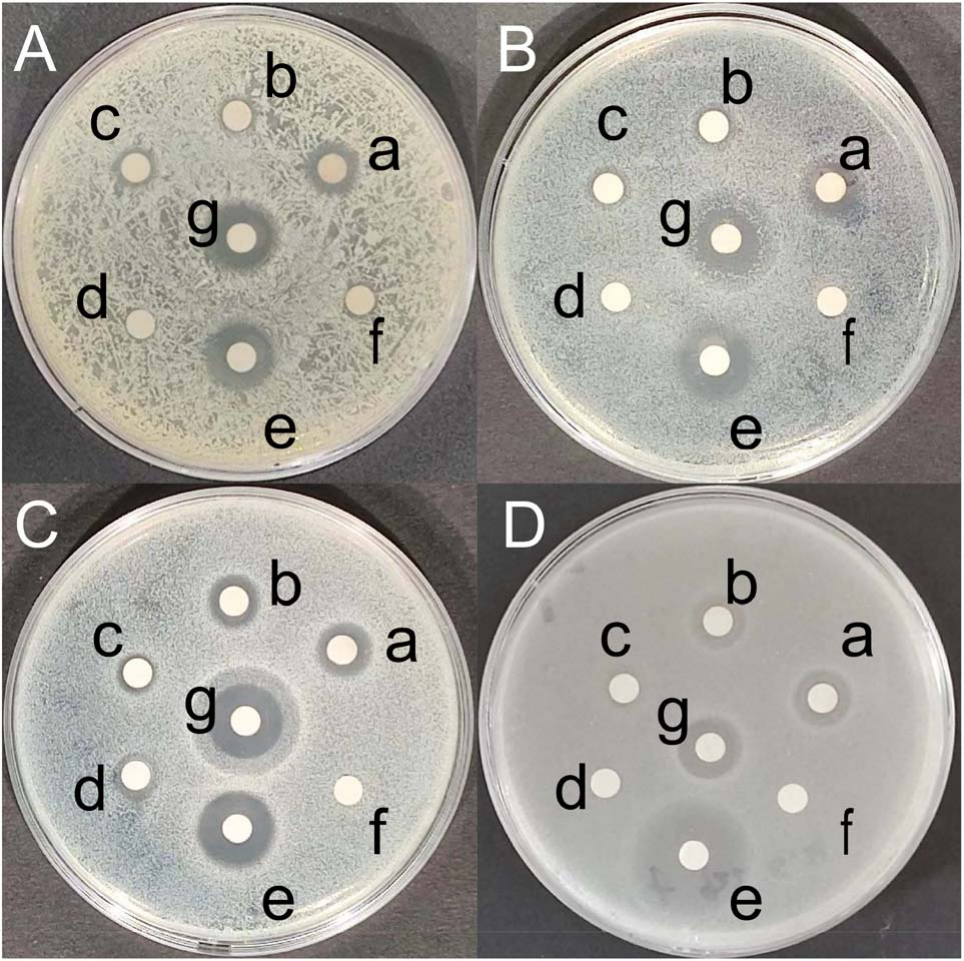
Bacterial growth-inhibition zones at different AgNP concentrations. (A) Leaf-AgNPs against *E. coli*. (B) Leaf-AgNPs against *S. aureus*. (C) Flower-AgNPs against *E. coli*. (D) Flower-AgNPs against *S. aureus*. (a) Plant extract; (b) 1 mM AgNO_3_; (c–f) 32, 64, 128, and 256 μg mL^−1^ of AgNPs, respectively; (g) ampicillin.

The disk diffusion assay results presented in Fig. 11 demonstrate that the pure extracts of *X. sorbifolia* leaves and flowers did not exhibit any antibacterial properties, indicating that the active antibacterial properties were primarily associated with the synthesized AgNPs. The antimicrobial efficacy of the AgNPs was observed to increase with their concentration. For leaf-AgNPs, inhibition zone diameters of 10.5 ± 0.2 mm and 10.4 ± 0.1 mm were observed against *S. aureus* and *E. coli*, respectively, at a concentration of 256 μg mL^−1^. Similarly, flower-AgNPs produced inhibition zone diameters of 10.5 ± 0.2 mm against *S. aureus* and 8.6 ± 0.7 mm against *E. coli*.

The comparable antibacterial properties of the leaf-AgNPs and flower-AgNPs may be attributed to the common bioactive constituents in the two plant extracts, which likely lead to the deposition of similar active compounds on the surface of the AgNPs. However, the slight variations in efficacy, particularly against *E. coli*, may be related to differences in the concentration and composition of phytochemicals between the plant extracts.

### 3.6 Cytotoxicity of AgNPs

#### 3.6.1. Cytotoxicity assessment

**Fig. 12:**
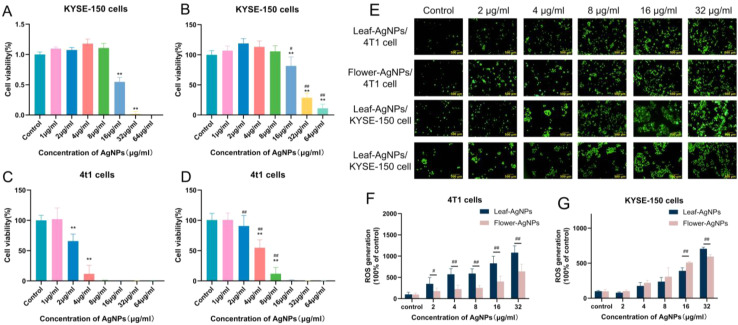
Anticancer effects of leaf-AgNPs and flower-AgNPs. (A–D) Cell viability of cancer cells treated with varying concentrations of AgNPs for 24 h and assessed using the CCK-8 assay. KYSE-150 cells treated with (A) leaf-AgNPs and (B) flower-AgNPs. 4T1 cells treated with (C) leaf-AgNPs and (D) flower-AgNPs. Data are presented as mean ± SD (*n* = 6) normalized to the untreated control group (100%). *p < 0.05, **p < 0.01 *versus* the untreated control group. ^#^*p* < 0.05 and ^##^*p* < 0.01 for comparisons between leaf-AgNP and flower-AgNP treatments at the same concentration. (E) Representative fluorescence microscopy images showing ROS generation (green DCFDA fluorescence) in 4T1 and KYSE-150 cells after treatment with AgNPs for 12 h. Scale bar = 500 μm. (F and G) Quantitative analysis of intracellular ROS levels after AgNP treatment. (F) ROS generation in 4T1 cells. (G) ROS generation in KYSE-150 cells. Data are presented as mean ± SD (*n* = 4) normalized to the untreated control group (set as 100%). ^#^*p* < 0.05, ^##^*p* < 0.01 for comparisons between leaf-AgNP and flower-AgNP treatments at the same concentration.

The cytotoxic effects of different concentrations of leaf-AgNPs and flower-AgNPs on the 4T1 and KYSE-150 cancer cell lines were evaluated using the CCK-8 assay, as illustrated in Fig. 12A–D. Both leaf-AgNPs and flower-AgNPs demonstrated a clear concentration-dependent cytotoxic effect against both cell lines. For instance, the treatment of KYSE-150 cells with leaf-AgNPs at a concentration of 32 μg mL^−1^ resulted in a significant reduction in cell viability to below 5% (Fig. 12A, *p* < 0.01 *vs.* control). Similarly, the exposure of 4T1 cells to leaf-AgNPs at 4 μg mL^−1^ led to a decrease in cell viability to approximately 11.81% (Fig. 12C, *p* < 0.01 *vs.* control). In comparison, flower-AgNPs at 64 μg mL^−1^ reduced KYSE-150 cell viability to approximately 11.99% (Fig. 12B, *p* < 0.01 *vs.* control), while at 8 μg mL^−1^, they decreased 4T1 cell viability to approximately 10% (Fig. 12D, *p* < 0.01 *vs.* control).

To quantitatively compare their cytotoxic potency, the half-maximal inhibitory concentration (IC_50_) values were determined by the interpolation of their dose-effect curves. For leaf-AgNPs, the IC_50_ values were 2.59 μg mL^−1^ against 4T1 cells and 17.45 μg mL^−1^ against KYSE-150 cells. For flower-AgNPs, the IC_50_ values were 4.65 μg mL^−1^ against 4T1 cells and 25.55 μg mL^−1^ against KYSE-150 cells. These IC_50_ values clearly indicate that the leaf-AgNPs possessed significantly higher cytotoxic potency (lower IC_50_) against both 4T1 and KYSE-150 cells than flower-AgNPs. Furthermore, both types of AgNPs demonstrated substantially stronger cytotoxic effects against 4T1 cells compared with KYSE-150 cells.

Further, statistical analysis was performed to directly compare the efficacy of leaf-AgNPs and flower-AgNPs at individual concentrations (Fig. 12A–D). Against KYSE-150 cells, leaf-AgNPs generally showed a higher cytotoxicity trend than flower-AgNPs across the tested concentrations. Leaf-AgNPs resulted in significantly lower cell viability than flower-AgNPs at 16 μg mL^−1^ (*p* < 0.05), 32 μg mL^−1^ (*p* < 0.01), and 64 μg mL^−1^ (*p* < 0.01) concentrations (comparing Fig. 12A and B). Against 4T1 cells, the superior potency of leaf-AgNPs, as indicated by its considerably lower IC_50_ value (2.59 μg mL^−1^*vs.* 4.65 μg mL^−1^ of flower-AgNPs), was further supported by direct comparisons at specific concentrations. The leaf-AgNPs also induced significantly lower cell viability than flower-AgNPs at 2, 4, and 8 μg mL^−1^ concentrations (*p* < 0.01) (comparing Fig. 12C and D). At higher concentrations (16 μg mL^−1^ and above), both leaf-AgNPs and flower-AgNPs induced near-complete cell death in 4T1 cells, with the cell viability approaching 0%.

In summary, based on both IC_50_ values and direct concentration-specific comparisons, leaf-AgNPs consistently demonstrated superior cytotoxic potency against both 4T1 and KYSE-150 cancer cell lines compared to flower-AgNPs. This enhanced efficacy was particularly evident against 4T1 cells at low to moderate concentrations at which significant differences were observed. Both AgNPs were notably more effective against 4T1 cells than KYSE-150 cells. These findings underscore the enhanced therapeutic potential of leaf-AgNPs in these cancer models.

#### 3.6.2. Measurement of ROS

The generation of ROS in response to AgNP treatment was quantified to investigate their role in mediating cytotoxicity effects, as illustrated in Fig. 12E (fluorescence microscopy) and Fig. 12F and G (quantitative analysis). A clear concentration-dependent increase in ROS production in 4T1 and KYSE-150 cancer cells was observed in the presence of leaf-AgNPs and flower-AgNPs in comparison with the control.

To elucidate the differential ROS-inducing capabilities, a direct statistical comparison between leaf-AgNPs and flower-AgNPs was performed at each tested concentration for both cell lines. In 4T1 cells, leaf-AgNPs consistently demonstrated a superior capacity to induce ROS generation compared to flower-AgNPs across the entire range of tested concentrations. In particular, leaf-AgNPs induced significantly higher levels of ROS than flower-AgNPs at a concentration of 2 μg mL^−1^ (*p* < 0.05), and this difference was even more pronounced at 4, 8, 16, and 32 μg mL^−1^ (*p* < 0.01 for each). This robust ROS induction by leaf-AgNPs aligns with its enhanced cytotoxic efficacy observed against 4T1 cells (Fig. 12G). In KYSE-150 cells, the relative ROS-inducing effects of the two AgNPs were more complex and concentration-dependent. At a concentration of 16 μg mL^−1^, the leaf-AgNPs generated significantly lower levels of ROS than flower-AgNPs (*p* < 0.01). Conversely, at 32 μg mL^−1^, the leaf-AgNPs induced significantly higher ROS production than flower-AgNPs (*p* < 0.01). At other tested concentrations (2, 4, 8, and 64 μg mL^−1^), no statistically significant differences in ROS generation were observed between the two types of AgNPs (Fig. 12F).

These results underscore the critical role of ROS generation in the anticancer mechanism of the synthesized AgNPs. Both types of AgNPs effectively stimulated ROS production in a concentration-dependent manner. However, their relative potency in inducing ROS and their potential contribution to cytotoxicity *via* this pathway varied significantly. This variation was dependent on the specific cancer cell line and nanoparticle concentration. Notably, the consistently higher ROS induction in 4T1 cells by leaf-AgNPs provides a strong mechanistic basis for its superior cytotoxic profile observed in this particular cancer cell model.

The cytotoxic effects of AgNPs may be mediated by various mechanisms, including the induction of structural damage to cancer cells, generation of ROS, DNA damage, protein deactivation, regulation of signalling pathways, and inhibition of cell migration and angiogenesis.^11^ Among these mechanisms, the primary driver of AgNP-induced toxicity is the generation of ROS, which leads to intracellular oxidative stress and triggers cell death.^62^ Previous studies have shown that the size and density of AgNPs significantly influence ROS production within cells.^63^ Smaller AgNPs with a higher surface-area-to-volume ratio lead to enhanced internalization and attachment to cells, thereby increasing ROS generation.^64^ In this study, the diameters of the synthesized AgNPs were found to be less than 40 nm, which correlates with their effective cytotoxic properties. A strong correlation was observed between AgNP concentration and cytotoxic efficacy, with higher concentrations resulting in a significant reduction in cancer cell viability. These findings are consistent with previous studies on AgNPs synthesized from the extracts of *Paeonia lactiflora* flowers and *Holigarna arnottiana*, which exhibited similar concentration-dependent cytotoxic activity.^64,65^ Additionally, the phytochemical capping of AgNPs derived from plant extracts might significantly enhance their anticancer properties. For instance, kaempferol is widely recognized for its ability to induce apoptosis in breast cancer cells.^66^ Polyphenols, such as epicatechin, detected in the leaf extracts are believed to possess unique anticancer properties.^67^ The phytochemical composition of the synthesized AgNPs likely influences their cytotoxicity toward the cancer cell lines, further underscoring the importance of the bioactive compounds in enhancing the anticancer efficacy of green-synthesized AgNPs. This study demonstrates AgNP-induced ROS generation. However, to unequivocally confirm the precise role of ROS in the observed cytotoxicity, further mechanistic validation is essential. Future studies using ROS scavengers such as *N*-acetylcysteine have been planned to address this, along with a detailed investigation of downstream apoptotic or mitochondrial pathways.^68^

Furthermore, while this study effectively demonstrates the cytotoxic potential of *X. sorbifolia*-derived AgNPs against the selected cancer cell lines, an important aspect for future investigation is their selectivity. Evaluating the effects of these AgNPs on non-cancerous (normal) cell lines will be crucial to determining their therapeutic index and assessing their safety profile. Such studies are essential before considering any potential translational applications of these AgNPs. Therefore, our future research is designed to provide a more comprehensive understanding of the biomedical potential of these green-synthesized nanoparticles.

Their biomedical potential can translate into several clinical applications. Their broad-spectrum antimicrobial activity, for example, suggests their utility in formulations, such as antimicrobial wound dressings. Moreover, their potent cytotoxic effects against cancer cells indicate potential for the development of topical agents for certain oncological therapies. However, it is crucial to acknowledge the path forward. While the AgNPs exhibited good colloidal stability and consistent bioactivity under the *in vitro* cell culture conditions used in this study, their behaviour and efficacy in more complex physiological environments are yet to be fully elucidated. Therefore, comprehensive investigations into their long-term stability, interaction with biological components (*e.g.*, serum, proteins), and sustained efficacy in such physiological milieus are essential. These studies will be paramount for assessing their true translational potential and paving the way for any clinical application.

## 4. Conclusions

In this study, AgNPs were synthesized using extracts derived from leaves and flowers of *X. sorbifolia*, a plant species prevalent in Northwest China. The leaf-AgNPs and flower-AgNPs synthesized in this work were small, spherical, and uniformly distributed, with an average radius of under 40 nm. Both AgNPs were enveloped by plant chemical constituents, due to which these AgNPs exhibited a synergistic effect. The synthesized AgNPs exhibited potent bactericidal activity against *E. coli*, although flower-AgNPs displayed inadequate efficacy against *S. aureus*. Overall, the AgNPs generated by plants exhibited potent cytotoxic activity against breast cancer cells at low concentrations. Of the two, Leaf-AgNPs exhibited superior cytotoxic efficacy. Furthermore, increasing the concentration of these particles also enhanced their efficacy against esophageal cancer cells. The cytotoxicity of the AgNPs corresponded with their ROS generation capability. To our understanding, this is the first report on AgNP synthesis using *X. sorbifolia* extracts. This method shows the potential to produce versatile AgNPs with antibacterial properties and cytotoxic effects against cancer cells, which makes them relevant in a wide range of clinical applications.

## Conflicts of interest

There are no conflicts to declare.

## Data Availability

All data generated or analyzed during this study are included in this published article.
